# Intravitreal aflibercept (Eylea) injection for cystoid macular edema secondary to retinitis pigmentosa - a first case report and short review of the literature

**DOI:** 10.1186/s12886-015-0033-z

**Published:** 2015-04-30

**Authors:** Giannis-Aimant Moustafa, Marilita M Moschos

**Affiliations:** Assistant Professor of Ophthalmology, University of Athens, Greece, 6 Ikarias Street, 14578 Ekali, Attica Greece; Department of Ophthalmology, Electrophysiology Laboratory, University of Athens, Athens, Greece

**Keywords:** Retinitis pigmentosa, Cystoid macular edema, Aflibercept, Eylea

## Abstract

**Background:**

Cystoid macular edema (CME) in retinitis pigmentosa (RP) has been managed in several ways as documented in the literature, with little success, though. The aim of our study was to report for the first time in literature the use of aflibercept in a patient with RP and CME.

**Case presentation:**

A 52-year-old man presented for blurred vision in his right eye. Best-corrected visual acuity (BCVA) was 3/10 in his right eye and 7/10 in his left eye. Physical examination and appropriate laboratory tests lead to the diagnosis of bilateral RP with CME in the right eye. Retinal thickness in the foveal area of the right eye was 631 μm. The patient was treated with a single intravitreal injection of 0.05 ml/0.5 mg aflibercept. One month later, BCVA of the right eye increased to 4/10, while BCVA of the left eye was unchanged. RT in the right eye decreased to 129 μm. Multifocal electroretinogram response did not improve, yet peaks were better-shaped and no areas of eccentral vision were present. Three and six months after injection, these improvements were maintained.

**Conclusion:**

This first-reported case indicates that intravitreal aflibercept injection for addressing CME in RP seems to be an effective treatment.

## Background

Retinitis pigmentosa (RP) is a group of inherited degenerative retinal disorders characterized by loss of photoreceptors (rods predominantly) and dystrophy of the pigment epithelium [1]. Visual acuity in RP patients is mostly deteriorated in the presence of cystoid macular edema (CME), an uncommon complication of the disease occurring in 10-20% of patients [2]. The pathogenesis of CME in RP is not clearly understood. Failure of the retinal pigment epithelium pumping mechanism and compromise of the blood-retinal barrier has been implicated [3,4]. Increased vascular permeability allows for fluid to accumulate in cystoid spaces within the retina [5].

Currently, there is no effective treatment suspending or reversing photoreceptor degeneration, yet complications, such as CME, have been managed in different ways. Some of the treatments that have been tested on CME in RP are systemic or topical carbonic anhydrase inhibitors (acetazolamide and dorzolamide, respectively), systemic or intravitreal (triamcinolone, dexamethasone) corticosteroids, laser photocoagulation and pars plana vitrectomy [2,4,6-21]. Some cohorts show favorable results with oral acetazolamide therapy achieving an increase in best-corrected visual acuity (BCVA), decrease in vascular leakage as seen with fluorescein angiography and decrease in retinal thickness [4,6-8]. Other studies failed to generate similar results [9,10], and indeed, in clinical practice, many are the patients who do not respond to this kind of treatment [22,23]. These unstable results in conjunction with the adverse effects of acetazolamide administration are the main reasons for which emergence of alternative therapies has become necessary. Only selected patients are benefited by intravitreal triamcinolone injection (IVT) and results seem to be short-lasting [2,15,16]. IVT may also cause glaucoma, cataract, infectious endophthalmitis and retinal detachment [16].

During the last seven years, attention has been given to the inhibition of the vascular endothelial growth factor (VEGF), when CME is present secondary to RP, which is known to promote endothelial cell mitosis and increase vascular permeability, resulting in edema [24].

In the context of these report studies with VEGF inhibitors for the management of CME associated with RP, we present for the first time in the literature a case of RP with CME treated with the anti-VEGF agent aflibercept (EYLEA; Regeneron Pharmaceuticals, Inc., Tarrytown, New York, USA and Bayer Healthcare Pharmaceuticals, Berlin, Germany).

## Case presentation

**Figure 1:**
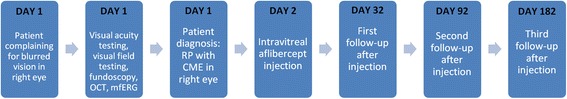
Timeline of events for the presented case.

**Figure 2:**
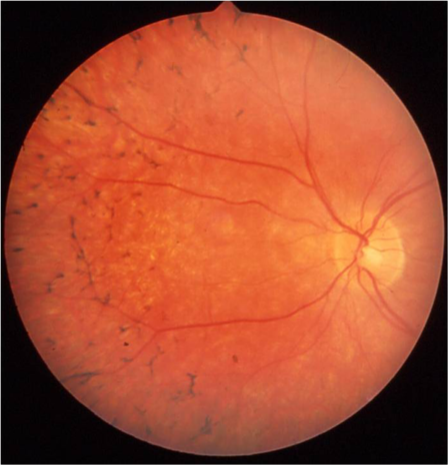
Fundoscopy showing peripheral bone-spicule-like pigmentations and arterial narrowing characteristic of retinitis pigmentosa.

**Figure 3:**
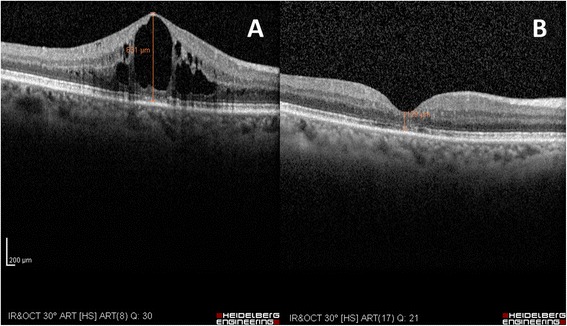
OCT scans of the right eye at presentation **(A)** and one month after intravitreal aflibercept **(B)** with macular thickness measuring 631 μm and 129 μm, respectively.

**Figure 4:**
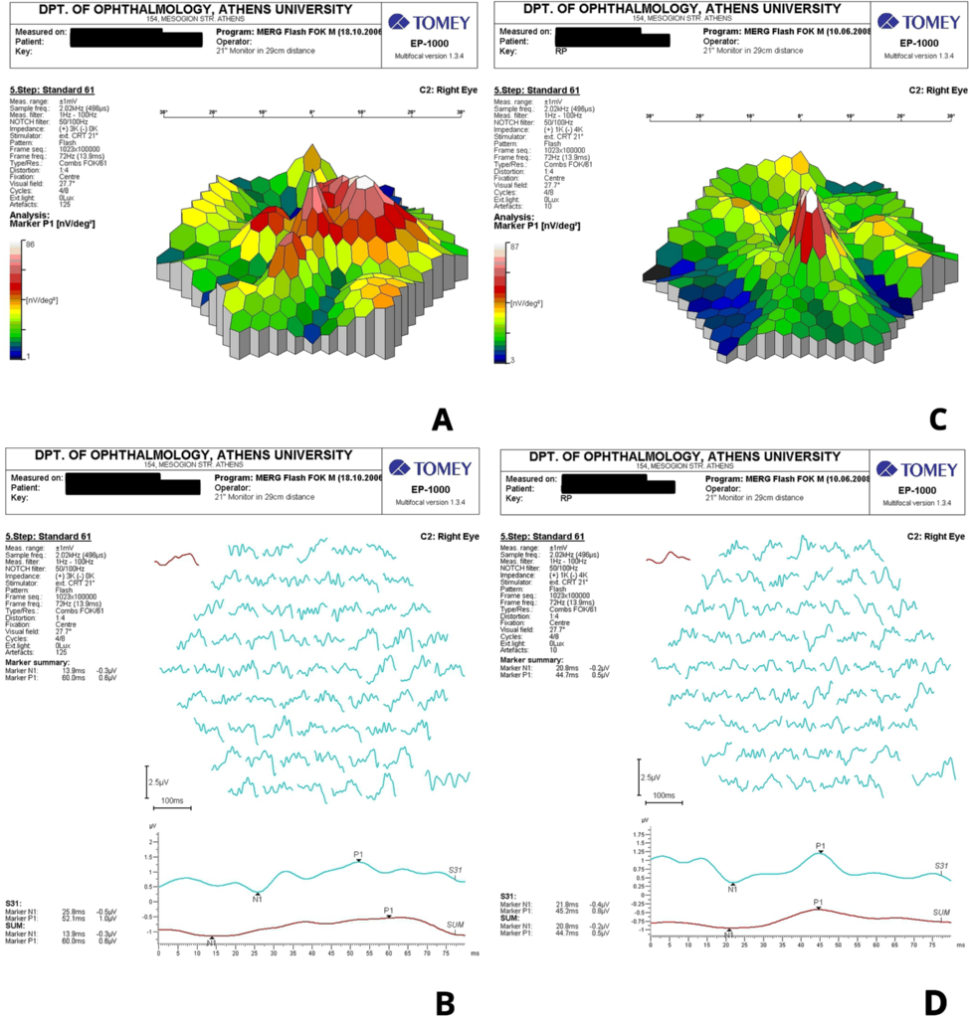
3D appearance of mfERG recording and mfERG traces on the right eye at presentation **(A,B)** and one month after the intravitreal aflibercept **(C,D)**.

A 52-year-old caucasian man (Figure 1) with no remarkable past medical and family history was referred to the First Department of Ophthalmology, University of Athens, Greece complaining for blurred vision in his right eye. Upon examination, BCVA was 7/10 in his left eye and 3/10 in his right eye. Fundoscopy of the left eye revealed mid-peripheral hyperpigmentary spots in form of bone-spicules and arteriolar narrowing, while in the right eye clinical findings were similar (Figure 2). Appropriate visual field tests, OCT and multifocal electroretinogram (mfERG) [25] were performed and, eventually, the patient was diagnosed with RP and CME of the right eye. OCT (Spectralis®, Track Laser Tomography) was performed to evaluate the macular edema. In addition to this, mfERG was performed to assess retinal function. OCT scan showed intraretinal fluid and a significant increase in retinal thickness in the foveal (631 μm) area (Figure 3). Moreover, mfERG showed decreased response in both eyes. The mean P1 retinal response density (RRD) amplitude of the foveal area was 86 nV/deg^2^ in right eye and 106 nV/deg^2^ in left eye (Figure 4).

Due to the controversial results of most therapeutic options for CME in RP (carbonic anhydrase inhibitors, corticosteroids), the patient was consulted to be treated with a VEGF inhibitor. He was informed about the off-label therapy and signed a written consent prior to therapy initiation. The VEGF inhibitor selected was aflibercept, due to its binding not only to the VEGF-A isomers (like bevacizumab and ranibizumab), but also to VEGF-B and placental growth factor [26]. Moreover, its longer duration of action and its powerful VEGF binding were additional factors that led to its selection [27]. A single injection of 0.05 ml/0.5 mg aflibercept (EYLEA; Regeneron Pharmaceuticals, Inc., Tarrytown, New York, USA and Bayer Healthcare Pharmaceuticals, Berlin, Germany) was performed to treat macular edema. One month after treatment, BCVA in the right eye elevated to 4/10 and macular edema had apparently improved, as it is depicted in the respective OCT scan (Figure 3). BCVA of the left eye was unchanged. Nevertheless, mfERG response remained decreased in both eyes (87 nV/deg^2^ in right eye and 106 nV/deg^2^ in left eye). It is worthy to say that even if the RRD remained decreased, the mfERG peaks were better shaped and there were not areas of eccentral vision in the one-month follow-up (Figure 4). The patient was also seen two months later (three months after treatment) with BCVA in his right eye still being 4/10. BCVA of the left eye was unchanged. At the last visit, six months after treatment, the improvement was maintained. No other drugs were co-administered and the patient did not receive any other kind of therapy for his condition.

## Discussion

CME is a complication of RP occurring in 10-20% of patients [2]. Currently there is not a gold-standard therapeutic option and treatment response seems to be individualized. Many caregivers deem carbonic anhydrase inhibitors to be the mainstay of treatment. Yet, many are the patients who fail to respond to carbonic anhydrase inhibitors therapy [9,10,22,23]. Additionally, considering their adverse effects, their use is discouraged. The same is true for IVT [2,15,16]. During the last few years, a limited amount of studies have tested VEGF inhibitors in the management of CME secondary to RP with generally good results [22,23,28]. In 2007, Melo et al. [29], presented two eyes of two different cases, in which intravitreal bevacizumab (AVASTIN; Genentech, South San Francisco, California, USA) was tested. The first patient failed to achieve an increase in visual acuity (VA, 2/200 pre- and post-injection) or decrease in retinal thickness (524 μm and 529 μm pre- and post-injection, respectively) after follow-up of one month, while in the second patient VA deteriorated from 2/100 to 2/200 and retinal thickness increased from 282 μm to 299 μm one month after the injection. Both patients showed ambiguous improvement with IVT. However, in 2009, Yuzbasioglu et al. [28] followed 13 eyes of 7 patients treated with intravitreal bevacizumab for a period of 10.3 months. VA increased from 5/400-20/100 to 20/200-20/63 and central macular thickness decreased from 245–603 μm to 124–168 μm. Other anti-VEGF agents have also been tested in CME secondary to RP, such as pegaptanib sodium (MACUGEN; EyeTech Pharmaceutical, Inc., New York, USA); particularly, Querques et al. [22] presented a case refractory to oral acetazolamide (1 month, 500 mg daily) with baseline BCVA 2/200 in his left eye (the one with the CME). One dose of intravitreal pegaptanib sodium 0.3 mg lead to improvement in BCVA to 20/40 and resolution of CME as seen with fundus microscopy and optical coherence tomography (OCT). Oral acetazolamide was continued for an additional month, and two months after acetazolamide withdrawal and 4 months after the pegaptanic sodium injection, BCVA still maintained at 20/40 and no recurrence of the CME was noticed. A cohort study reporting on the use of anti-VEGF agents in CME-RP was conducted in 2009 by Artunay et al. [23], who used intravitreal ranibizumab (LUCENTIS; Genentech, South San Francisco, California, USA) in 15 eyes of 15 patients with persistent edema at least 6 months despite acetazolamide therapy. 15 eyes of 15 similar patients who refused this off-label treatment were considered the control group. 6 months after one dose of intravitreal ranbizumab 0.5 mg, both central foveal thickness and BCVA showed improvement, although improvement of BCVA was not statistically significant (p > 0.05). However, none of the studies above used aflibercept for this condition and none of the studies used multifocal-electroretinogram to assess retinal function.

Aflibercept is a chimeric protein consisted of the extracellular portion of the human VEGF receptors 1 and 2 (binding section) and the Fc portion of IgG1 immunoglobulin [30]. This structure ensures a very high VEGF binding affinity [27]. Furthermore, its long duration of action makes it an interesting new agent, since it may reduce frequency of repeat injections if needed, and establish a more durable effect [26,27]. Most frequent adverse reactions (≥5%) that have been observed with intravitreal aflibercept injection are conjunctival hemorrhage, eye pain, cataract, vitreous floaters, and transiently increased intraocular pressure [31]. Severe adverse events, such as endophthalmitis and retinal detachment are rare (<0.1%) [31]. Currently, aflibercept has been approved by the US Food and Drug Administration (FDA) for the management of neovascular (wet) age-related macular degeneration (2011), diabetic macular edema (2014) and macular edema following retinal vein occlusion (2014). No testing has been made on macular edema associated with RP and this case report is the first clue of efficacy of aflibercept. Our patient showed a 10% improvement in BCVA and significant resolution of macular edema in OCT scan, although mfERG response remained decreased (yet, better-shaped peaks and no areas of eccentral vision).

Our results show that there are anatomical correlates to support the concept of macular edema amelioration. This is mainly the prominent decrease of macular thickness as measured by OCT. The mean visual acuity improved only by 10% one month after treatment and remained unchanged two months later. On the contrary, mfERG findings did not follow the decrease of macular thickness. These findings show that the increase of visual acuity, as also the improvement of electrical responses of the macular area did not follow the decrease of macular thickness. This may be explained by the fact that macular edema is only a parameter that may affect visual acuity and electrophysiological responses in the beginning of the disease. Atrophy of the retina, particularly of the photoreceptors, atrophy of the pigment epithelium and scarring are all unmeasured variables, which have potential to impact vision. Of course, we are not able to definitely conclude on the issue, merely based on this case and further studies with larger samples and a longer follow-up period are needed for this reason. However the anatomical improvement is very promising and further investigation must be done.

## Conclusion

Intravitreal aflibercept injection treatment seems to be efficacious for addressing CME secondary to RP. Additional studies are needed to confirm the aforementioned result.

## Consent

Written informed consent was obtained from the patient for publication of this Case report and any accompanying images. A copy of the written consent is available for review by the Editor of this journal.

## Abbreviations

BCVA: Best-corrected visual acuity
CME: Cystoid macular edema
IVT: Intravitreal triamcinolone
mfERG: Multi-focal Electroretinogram
OCT: Optical coherence tomography
RP: Retinitis pigmentosa
RRD: Retinal response density
VA: Visual acuity
VEGF: Vascular endothelial growth factor
